# Self-care practices and associated factors among adult hypertensive patients in Ayder Comprehensive Specialized Hospital, Tigray, Ethiopia, 2018

**DOI:** 10.1186/s13104-019-4502-y

**Published:** 2019-08-06

**Authors:** Gebrewahd Bezabh Gebremichael, Kalayou Kidanu Berhe, Birhane Gebrehiwot Beyene, Kiros Belay Gebrekidan

**Affiliations:** School of Nursing College of Health Science, Mekelle Univesity, Mekelle, Tigray Ethiopia

**Keywords:** Self-care, Hypertension, Ethiopia

## Abstract

**Objectives:**

To assess self-care practices and associated factors among hypertensive patients in Ayder Comprehensive Specialized Hospital 2017/2018.

**Result:**

Good self-care practice was found only among 20.3% of respondents. Adherence to not smoking, anti-hypertensive medication, alcohol abstinence, dietary management, physical exercise and weight management was found to be 99.1%, 74.10%, 67.20%, 63.10%, 49.4% and 40.6% respectively. Sex (AOR = 2.254, 95% CI 1.092–4.653), age (AOR = 3.265, 95% CI 1.030–10.355), educational status (AOR = 4.205, 95% CI 1.304–13.559), disease duration (AOR = 3.124, 95% CI 1.204–8.105), BP status (AOR = 2.728, 95% CI 1.256–5.926) and knowledge (AOR = 6.196, 95% CI 2.906–13.214) showed significant statistical association with self-care practice.

## Introduction

Hypertension known as “the silent killer” is a major public health problem both in developed and developing countries [1]. It affects 22% of the world’s population aged ≥ 18 years [2] and 1.13 billion adults [3] and this number is expected to increase to 1.56 billion by 2025 [4]. Hypertension is the leading cause of death and disability accounting for 9.4 million annual deaths [5].

Hypertension related death and disability stem primarily from cardiovascular disease (CVD), renal failure and dementia [6]. For every 20 mmHg increase in systolic blood pressure (BP) to > 115 mmHg or 10 mmHg increase in diastolic BP to 75 mmHg, the risk of cardiovascular and stroke events doubles [7]. Besides, hypertension costs about 10% of the global health expenditure [8].

Self-care practices have been proved as the determinants of hypertension prevention and control [9–11]. Major self-care practices for hypertension include medication adherence, maintenance of healthy body weight (body mass index (BMI) 18.5 to 24.9, and waist circumference 102 cm for men and 88 cm for women), 30–60 min of moderate intensity exercise 4–7 days/week in addition to the routine activities, dietary approach to stop hypertension (DASH) and low salt diet, moderation of alcohol consumption (men ≤ 2 and women ≤ 1 alcoholic drink), cessation of smoking and stress management [10–13]. Hypertensive clients should also monitor their BP [14].

Despite the availability of anti-hypertensive drugs and optimal hypertension self-care practices are proved to decrease BP and all cause mortality rate and cardio-vascular morbidities [15, 16], hypertensive patients are suffering from unfavorable self-care practices. Many studies have shown that hypertensive patients have poor self-care practices [17–21].

World health organization (WHO) develops Global Action Plan on; (1) 25% reduction in non-communicable diseases (NCDs); (2) 10% reduction in alcohol use; (3) 10% reduction in insufficient physical activity; (4) 30% reduction in mean population salt intake; (5) 30% reduction in tobacco use; (6) 25% reduction in prevalence of raised BP; (7) 0% increase in obesity and diabetes; (8) at least 50% rate of eligible people receiving drug therapy to prevent heart attack and stroke to be achieved by 2025 using the year 2010 as the baseline [22].

To achieve this, cost-effective and research supported health care services drawing on the best available evidence are essential. Thus, the purpose of this study is to assess self-care practices and associated factors among hypertensive patients.

## Main text

### Methods

Study area and period: The study was conducted in Ayder Comprehensive Specialized Hospital (ACSH), Tigray, Ethiopia [23] from November 2017 to June 2018.

Study design and population: Hospital-based cross-sectional study design was conducted. All adult hypertensive patients who had at least 6 months follow-ups were included as a source population. However unconscious and critical ill patients and pregnant mother were excluded.

Sample size determination: Sample size was determined using single population proportion formula considering: Prevalence (p) of lifestyle modification practice 0.273 [21] 95% confidence interval and 5% margin of error. Adding 5% none response rate the total sample size was 320.

Sampling procedure and techniques: Sampling frame was created and participants were selected using simple random sampling technique.

#### Study variables

Independent variables: socio-demographic variables (age, sex, income, educational status, occupation and residence), health profile of patients (family history, BMI, BP monitoring, BP status, co-morbidity, disease duration and number and type of medication) and knowledge of hypertension and its management.

Dependent variable: self-care practice.

Data collection tool: data were collected using interviewer administered structured questionnaire. Self-care practice was assessed using Hypertension Self-Care Activity Level Effects (H-SCALE) which includes medication adherence, dietary management, smoking status, physical activity, weight management and alcohol intake. H-SCALE has provided detailed description of the scoring method of each component [24]. Hypertension evaluation of lifestyle and management (HELM) scale was used to assess respondents’ knowledge [27].

#### Operational definitions

Good self-care practice: adherence to all the components of the H-SCALE.

Poor self-care practice: non-adherence to at least one component of the H-SCALE.

Good knowledge: scores of above mean value on HELM scale.

Poor knowledge: scores of below mean value on HELM scale.

Data quality assurance: training was given to data collectors and supervisors. Pretest was conducted in 5% of the samples in Axum comprehensive specialized hospital. Supervision was made, and data were checked at the spot.

Data processing and analysis: data were entered to EpiInfo and exported to SPSS for analysis. Descriptive statistics was done and binary logistic regression model was used to identify the associated factors. Significance was declared using adjusted odds ratios with 95% confidence interval (CI) at p*-*value < 0.05.

### Result

#### Socio-demographic characteristics

A total of 320 respondents (100% response rate) were interviewed. Around 51.2% were females. The mean age of the respondents was 53.83 ± 14.52 years with a minimum age of 19 and a maximum age of 85 years. Majority of the respondents (70.9%) were < 65 years old. One hundred thirty-eight (43.1%) of the respondents were not able to read and write (Table 1).

**Table 1:** Sociodemographic characteristics of hypertensive patients at ACSH, Mekelle, Tigray, Ethiopia, 2018 (n = 320)

Variables	Category	Good practice N (%)	Poor practice N (%)	Total N (%)
Sex	Male	18 (5.6)	138 (43.1)	156 (48.8)
	Female	47 (14.7)	117 (37.2)	164 (51.2)
Age	< 65	60 (18.8)	166 (51.9)	226 (70.6)
	≥ 65	5 (1.6)	89 (27.8)	94 (29.4)
Educational status	Can’t read an d write	12 (3.8)	125 (39.1)	137 (42.8)
	Can read and write	8 (2.5)	46 (14.4)	54 (16.9)
	Primary	3 (0.9)	19 (5.9)	22 (6.9)
	Secondary	6 (1.9)	12 (3.8)	18 (5.6)
	Collage and above	36 (11.2)	53 (16.6)	89 (27.8)
Occupation	Farmer	9 (2.8)	46 (14.4)	55 (17.2)
	Civil servant	16 (5)	48 (15)	64 (20)
	House wife	8 (2.5)	23 (7.2)	31 (9.7)
	Self employed	21 (6.6)	65 (20.3)	86 (26.9)
	Pensioned	1 (0.3)	3 (0.9)	4 (1.2)
	Unemployed	7 (2.2)	63 (19.7)	70 (21.9)
	Others	3 (0.9)	7 (2.2)	10 (3.1)
Income	< 500	2 (0.6)	20 (6.2)	22 (6.9)
	500–1000	16 (5)	105 (32.8)	121 (37.8)
	> 1000	45 (14.1)	132 (41.2)	177 (55.3)
Residence	Urban	54 (16.9)	178 (55.6)	232 (72.5)
	Rural	9 (2.8)	79 (24.7)	88 (27.5)

#### Health profile related factors

Ninety-seven (30.3%) respondents had family history of hypertension. The mean duration of hypertension was 3.50 ± 3.07 with a range of 0.5–20 years. Majority of respondents (67.2%) had normal BMI and 50.3% of respondents had medically confirmed co-morbidity. Most of the respondents (76.2%) had ≥ 140/90 mmHg average BP of three consecutive follow-ups. Ninety-six respondents (30%) had ever missed follow-ups. Majority of respondents (71.2%) was taking ≤ 2 types of anti-hypertensive medications (Table 2).

**Table 2:** Distribution of health profile of hypertensive patients at ACSH, Mekelle, Tigray, Ethiopia 2018 (n = 320)

Variables	Category	Good practice N (%)	Poor practice N (%)	Total N (%)
Family history	Yes	25 (7.8)	72 (22.5)	97 (30.3)
	No	38 (11.9)	185 (57.8)	223 (69.7)
Disease duration (years)	< 2	11 (3.4)	63 (19.7)	74 (23.1)
	2–4	21 (6.6)	113 (35.3)	134 (41.9)
	≥ 4	31 (9.7)	81 (25.3)	112 (35)
BMI	Under-weight	3 (0.9)	13 (4.1)	16 (5)
	Normal	4 (12.8)	174 (54.4)	215 (67.2)
	Over-weight	17 (5.3)	65 (20.3)	82 (25.6)
	Obesity	2 (0.6)	5 (1.6)	7 (2.2)
Co-morbidity	Yes	34 (10.6)	127 (39.7)	162 (50.3)
	No	29 (9.1)	130 (40.6)	159 (49.7)
BP status	Controlled	95 (7.8)	51 (15.9)	76 (23.8)
	Uncontrolled	38 (11.9)	206 (64.4)	244 (76.2)
Regular BP check (twice a month)	Yes	34 (10.6)	103 (32.2)	137 (42.8)
	No	31 (9.7)	152 (47.5)	183 (57.2)
Miss follow up	Yes	11 (3.4)	85 (26.6)	96 (30)
	No	52 (16.2)	172 (53.8)	224 (70)
Number of type of medication	≤ 2	49 (15.3)	179 (55.9)	228 (71.2)
	≥ 3	14 (4.4)	78 (24.4)	92 (28.8)

#### Information and knowledge

Most of the respondents (91.9%) got information about hypertension and its management. The mean score of knowledge was 4.3. Nearly 45.6% of participants had good knowledge and 33.56% of them had good self-care practice. However, only 9.1% of participants with poor knowledge had good self-care practice.

#### Self care practice

Good self-care practice was found only among 20.3% [95% CI 15.9–24.7] of respondents. Around 99.1% of respondents were non-smokers. Nearly three-fourth (74.10%) of participants were adherent to anti-hypertensive medication. More than two-third (67.20%) of participants were non-drinkers. Adherence to dietary management, physical exercise and weight management were found to be 63.10%, 49.4% and 40.6% respectively.

#### Factors associated with self-care practice

Sex, age, educational status, monthly income, residency, family history, disease duration, BP status, BP check up, follow up miss, number and type of medication, information and knowledge were significant in the bivariate logistic regression. However, in the multivariable binary logistic regression analysis, only sex, age, educational status, disease duration, BP status and knowledge were independent predictors of self-care practices.

Females were found 2.254 times more likely to have good self-care practice than males (AOR = 2.254, 95% CI 1.092–4.653, p = 0.028). Patients aged < 65 years old were 3.265 times more likely to have good self-care practice than patients ≥ 65 years old (AOR = 3.265, 95% CI 1.030–10.355, p = 0.044). Having educational status of college and above was found to be 4.205 times more associated to good self-care practice than unable to read and write [AOR = 4.205, 95% CI 1.304–13.559, p = 0.016).

Respondents with ≥ 4 years of disease duration were 3.124 times more likely to practice good self-care as compared to those with < 2 years of disease duration (AOR = 3.124, 95% CI 1.204–8.105, p = 0.019). Controlled BP was found 2.7 times more associated to good self-care practice than uncontrolled BP (AOR = 2.728, 95% CI 1.256–5.926, p = 0.011). Good knowledge was found 6.196 times more positively associated to good self-care practice than poor knowledge (AOR = 6.196, 95% CI 2.906–13.214, p = 0.000) (Table 3).

**Table 3:** Bivariate and multivariable logistic regression analysis result for significant variables among hypertensive attending at ACSH, Mekelle, Tigray Region, Ethiopia 2018 (n = 320)

Variables	Category	Good practice N (%)	Poor practice N (%)	COR	AOR	p-value
Sex	Male	18 (5.6)	138 (43.1)	1	1	
	Female	47 (14.7)	117 (36.6)	3.08 (1.696–5.592)	2.254 (1.092–4.653)**	0.028
Age	< 65	60 (18.8)	166 (51.9)	6.434 (2.493–16.602)	3.265 (1.030–10.355)**	0.044
	≥ 65	5 (1.6)	89 (27.8)	1	1	
Educational status	Can’t read $ write	12 (3.8)	125 (39.1)	1	1	
	Can read $ write	8 (2.5)	46 (14.4)	1.812 (0.696–4.714)	1.228 (0.353–4.278)	0.747
	Primary	3 (0.9)	19 (5.9)	1.645 (0.425–6.370)	1.174 (0.211–6.538)	0.854
	Secondary	6 (1.9)	12 (3.8)	5.208 (1.657–16.368)	4.131 (0.861–19.813)	0.076
	Collage $ above	36 (11.2)	53 (16.6)	7.075 (3.416–14.653)	4.205 (1.304–13.559)**	0.016
Income	< 500	2 (0.6)	20 (6.2)	1	1	
	500–1000	16 (5)	105 (32.8)	1.524 (0.325–7.149)	2.205 (0.316–15.402)	0.425
	> 1000	45 (14.1)	132 (41.2)	3.615 (0.814–16.063)	2.646 (0.375–18.666)	0.329
Residence	Urban	56 (17.5)	176 (55)	2.793 (1.316–5.926)	1.101 (0.354–3.420)	0.868
	Rural	9 (2.8)	79 (24.7)	1	1	
Family Hx	Yes	25 (7.8)	72 (22.5)	1.728 (0.980–3.045)	1.346 (0.632–2.865)	0.441
	No	38 (11.9)	185 (57.8)	1	1	
Disease duration (years)	< 2	11 (3.4)	63 (19.7)	1	1	
	2–4	21 (6.6)	113 (35.3)	1.015 (0.470–2.189)	0.563 (0.218–1.454)	0.235
	≥ 4	31 (9.7)	81 (25.3)	1.977 (0.940–4.161)	3.124 (1.204–8.105)**	0.019
BP status	Controlled	95 (7.8)	51 (15.9)	2.733 (1.523–4.905)	2.728 (1.256–5.926)**	0.011
	Uncontrolled	38 (11.9)	206 (64.4)	1	1	
BP check	Yes	34 (10.6)	103 (32.2)	1.619 (0.936–2.798)	0.7 (0.318–1.541)	0.375
	No	31 (9.7)	152 (47.5)	1	1	
Miss follow up	Yes	11 (3.4)	85 (26.6)	1	1	
	No	52 (16.2)	172 (53.8)	2.455 (1.221–4.936)	1.508 (0.628–3.618)	0.358
No $ type medication	≤ 2	49 (15.3)	179 (55.9)	1.605 (0.839–3.071)	1.203 (0.511–2.836)	0.672
	≥ 3	14 (4.4)	78 (24.4)	1	1	
Information	Yes	63 (19.7)	231 (72.2)	3.273 (0.753–14.222)	2.880 (0.488–16.990)	0.243
	No	2 (0.6)	24 (7.5)	1	1	
Knowledge	Good	49 (15.3)	97 (30.3)	4.988 (2.688–9.258)	6.196 (2.906–13.214)**	0.000
	Poor	16 (5)	156 (49.4)	1		

**AOR is significant at p-value < 0.05

### Discussion

Good self-care practice was found only among 20.3% [95% CI 15.9–24.7] of the respondents. This finding is consistent with the studies conducted in Addis Ababa (23%) [20] and Nigeria (16.4%) [19]. However, this finding is lower than the study conducted in south Ethiopia (27.3%) [21] and India (37.1%) [25]. This could be explained by socio-cultural variation that influences life style of individuals, sample size difference and variation in the components of self-care practice assessment tool.

Being female was 2.25 [AOR = 2.25, 95% CI 1.09–4.65, p = 0.028] more likely associated with good self-care practice. This is in line with the study conducted in Addis Ababa [20]. This consistency could be because of females are more adherent to the most components of the H-SCALE. It could also be due to socio-environmental and cultural reasons i.e. females may not smoke or use alcohol not essentially due to their hypertensive condition but could be attributed to societal culture.

Respondents < 65 years old had 3.26 [AOR = 3.26, CI 95, 1.03–10.35, p = 0.044] more good self-care practice than ≥ 65 years old respondents. This finding was in line with the study conducted in India which showed older age is associated with unfavorable self-care practice [25]. This was supported by the study conducted in South Ethiopia which showed patients aged ≥ 65 years were less likely to have good lifestyle modification practice than patients with < 65 years [21]. But this study finding is in contradict to the study finding conducted in Addis Ababa and Israel which showed younger age is less likely associated with self-care practice [20, 26]. This might be due to the difference in participants’ age categorization and sample size.

Respondents who had an educational status of college and above had 4.21 [AOR = 4.21, 95% CI 1.304–13.559, p = 0.016] more good self-care practice than those who cannot read and write. This is in line with the study conducted in India [17]. Having good educational status helps individuals to have good knowledge and thus promotes respondents’ good self-care practices.

Greater than 4 years of disease duration was 3.12 [AOR = 3.12, 95% CI 1.20–8.10, p = 0.019] more likely associated with good self-care practice than < 2 years of disease duration. This is supported by the studies conducted Addis Ababa [20] and South Ethiopia [21] which showed that patients with longer disease duration had good life-style modification practice. This consistency could be due to the continued counseling’ and health education on self-care practice.

Controlled BP was found 2.73 [AOR = 1.25.95% CI 5.92, p = 0.011] more likely associated with good self-care practice. This might be because of hypertension is essentially controlled and reduced by good self-care practices.

Good knowledge was 6.19 times more associated to good self care practice [AOR = 6.19, 95% CI 2.9–13.2, p = 0.000]. This is also backed by the study conducted in Addis Ababa [20]. This similarity could be due to having good knowledge promote patients to give more emphasis on self-care practice.

## Limitations

There may have been recall bias and social desirability bias since the self-care practices of the study participants were based on self-reports. There are no adequate similar studies on self-care practice and most literatures used different statistical analysis. Thus, comparisons were difficult in self-care practices.

## Data Availability

Raw data can be made available on request to the corresponding author.

## Abbreviations

ACSH: Ayder Comprehensive Specialized Hospital
AOR: adjusted odds ratio
COR: crude odds ratio
BMI: body max index
BP: blood pressure
CI: confidence interval
CVD: cardiovascular disease
DASH: Dietary Approaches to Stop Hypertension
HELM: hypertension evaluation of lifestyle and management
HRERC: Health Research Ethics Review Committee
H-SCALE: Hypertension Self-Care Activity Level Effect
NCD: non-communicable disease
SPSS: Statistical Package for Social Sciences
WHO: World Health Organization
